# The impact of disease-related knowledge on perceptions of stigma among patients with Hepatitis C Virus (HCV) infection

**DOI:** 10.1371/journal.pone.0258143

**Published:** 2021-10-05

**Authors:** M. Elle Saine, Julia E. Szymczak, Tyler M. Moore, Laura P. Bamford, Frances K. Barg, Kimberly A. Forde, Jason Schnittker, John H. Holmes, Nandita Mitra, Vincent Lo Re

**Affiliations:** 1 Department of Biostatistics, Epidemiology, and Informatics, Perelman School of Medicine, University of Pennsylvania, Philadelphia, PA, United States of America; 2 Leonard Davis Institute of Health Economics, University of Pennsylvania, Philadelphia, PA, United States of America; 3 Department of Psychiatry, Brain Behavior Laboratory, Perelman School of Medicine, University of Pennsylvania, Philadelphia, PA, United States of America; 4 Division of Infectious Diseases and Global Public Health, Department of Medicine, University of California San Diego School of Medicine, San Diego, California, United States of America; 5 Department of Family Medicine and Community Health, University of Pennsylvania Perelman School of Medicine, Philadelphia, PA, United States of America; 6 Section of Hepatology, Lewis Katz School of Medicine at Temple University, Philadelphia, PA, United States of America; 7 Department of Sociology, University of Pennsylvania, Philadelphia, PA, United States of America; 8 Division of Infectious Diseases, Department of Medicine, Perelman School of Medicine, University of Pennsylvania, Philadelphia, PA, United States of America; University of Washington, UNITED STATES

## Abstract

Most patients with hepatitis C virus (HCV) infection perceive some degree of disease-related stigma. Misunderstandings about diseases may contribute to disease-related stigma. The objective of this study was to evaluate patient-level knowledge about HCV infection transmission and natural history and its association with HCV-related stigma among HCV-infected patients. We conducted a cross-sectional survey study among 265 patients with HCV in Philadelphia using the HCV Stigma Scale and the National Health and Nutrition Examination Survey (NHANES) Hepatitis C Follow-up Survey (2001–2008). The association between HCV knowledge and HCV-related stigma was evaluated via linear regression. Overall knowledge about HCV transmission and natural history was high, with >80% of participants answering ≥9 of 11 items correctly (median number of correct responses, 9 [82%]), HCV-related knowledge was similar between HIV/HCV-coinfected and HCV-monoinfected participants (p = 0.30). A higher level of HCV-related knowledge was associated with greater perceived HCV-related stigma (β, 2.34 ([95% CI, 0.51–4.17]; p = 0.013). Results were similar after adjusting for age, race, ethnicity, HIV status, education level, stage of HCV management, time since diagnosis, and history of injection drug use. In this study, increased HCV-related knowledge was associated with greater perceptions of HCV stigma. Clinicians may consider allotting time to address common misconceptions about HCV when educating patients about HCV infection, which may counterbalance the stigmatizing impact of greater HCV-related knowledge.

## Introduction

Misinformation about diseases can engender fear at both the individual and population levels, contributing to the persistence of disease-based stigma [1]. Stigma has been identified as an important mediator of health behaviors, such as disease disclosure, treatment uptake, and medication adherence [1–4]. Previously, we found that 95.5% of patients with hepatitis C virus (HCV) infection experience some degree of perceived disease-related stigma from others [5]. Significant HCV-related misconceptions and knowledge gaps are prevalent among persons with HCV infection [6–8]. Studies of HIV-related stigma have previously reported that less knowledge about HIV infection is associated with greater perceived stigma [2, 3]. However, the association between HCV-related knowledge and perceptions of stigma remains unknown among persons with HCV infection. Addressing deficits in HCV-related knowledge may ameliorate HCV-associated stigma and lead to increased engagement of patients in the steps across the HCV care continuum.

We evaluated patient-level knowledge about HCV infection transmission and natural history and its association with HCV-related stigma. We hypothesized that less knowledge about HCV infection would be associated with greater perceived HCV stigma.

## Materials and methods

### Study design and setting

Between July 2018 and May 2019, we conducted a cross-sectional study among English-speaking adults aged ≥18 years with a history of HCV infection (defined by positive HCV antibody). By including patients with a positive HCV antibody, we were able to include patients who had ever been diagnosed with HCV infection, including patients who have chronic HCV, spontaneously cleared the virus, or were cured with antiviral treatment. Participants were enrolled at five outpatient infectious disease practices across two Philadelphia health systems: University of Pennsylvania Health System and Philadelphia Field Initiation Group for HIV Trials (FIGHT) Community Health Centers. One of the participating practices was housed within a syringe service program where patients were also undergoing active treatment for substance use disorder. All participants provided verbal informed consent before participating in the study. The study setting, participant recruitment, and procedures have been previously described [5, 9]. This study was approved by the Institutional Review Boards of the University of Pennsylvania and Philadelphia FIGHT.

### Data collection

Two self-administered surveys were completed by HCV antibody-positive participants on laptop computers equipped with headphones and audio-computer assisted self-interview software (ACASI).

HCV-related knowledge was measured using the National Health and Nutrition Examination Survey (NHANES) Hepatitis C Follow-up Survey (2001–2008), composed of 11 statements designed to test knowledge about HCV-related transmission and natural history (questions appear in **Table 1**) [6]. Eight of the questions are true statements about HCV infection, while three are false. Using the scoring methods employed during NHANES administration [6], an entry was assigned “1” if answered correctly and “0” if answered incorrectly, declined, or answered as “Don’t know.”

**Table 1:** Baseline characteristics of participants.

Characteristics	Overall
	(n = 265)
**Age, years (n, %)**	
18–34	37 (13.96%)
35–44	63 (23.77%)
45–54	71 (26.79%)
55+	94 (35.47%)
**Sex/gender identity (n, %)**	
Male	182 (68.68%)
Female	75 (28.30%)
Transgender male to female	4 (1.51%)
Transgender female to male	2 (0.75%)
Queer / Non-binary / Gender non-conforming / Prefer not to answer	2 (0.75%)
**Race (n, %)**	
White	124 (46.79%)
Black / African American	104 (39.25%)
Multiracial / Other / Prefer not to answer	37 (13.96%)
**Ethnicity (n, %)**	
Not Hispanic / Latinx	205 (77.36%)
Hispanic / Latinx	57 (21.51%)
**Education (n, %)**	
Less than high school degree	59 (22.35%)
High school degree / GED	130 (49.24%)
Associated degree / Some college	55 (20.83%)
College Degree	20 (7.58%)
**HIV co-infection**	147 (55.47%)
**Time since HCV diagnosis (n, %)**	
Diagnosed within previous year	37 (14.68%)
1–5 years	75 (29.76%)
> 5 years	140 (55.56%)
**Stage of HCV management (n, %)**	
Spontaneously cleared	13 (4.91%)
Diagnosed, Untreated	48 (18.11%)
Previously treated, Not cured	17 (6.42%)
Currently being treated	77 (29.06%)
Treated, Cured	110 (41.51%)
**History of injection drug use**	196 (73.96%)

Abbreviations: GED = General Educational Development; HIV = Human Immunodeficiency Virus; HCV = Hepatitis C Virus.

HCV-related stigma was measured using the validated 33-item HCV Stigma Scale (HCV-SS) [9]. Item responses were recorded on a four-point Likert scale ranging from 1 (strongly disagree) to 4 (strongly agree) and item scores were summed to achieve the total HCV-SS score (range, 33–132), with higher scores indicating greater perceived stigma. Additionally, we collected self-reported demographic information, education level, history of injection drug use, and clinical data, including HIV status, time since HCV diagnosis, and stage of HCV management.

### Statistical analysis

For the knowledge survey, the median number of correct responses, with interquartile ranges (IQRs), were calculated. We constructed linear regression models to determine the association between HCV-related stigma (outcome variable) and the total number of correctly answered responses on the NHANES knowledge survey (independent variable). To assess whether this association differed by HIV status, we examined the interaction between HIV-coinfection status and number of correct responses to knowledge items.

Multivariable linear regression was performed, adjusting for age, race, ethnicity, HIV status, education level, stage of HCV management, time since diagnosis, and history of injection drug use. To account for potential affirmative response bias, we conducted a sensitivity analysis excluding participants who responded “True” to all knowledge items.

For individual knowledge items, the frequency of correct responses was calculated and the association with stigma was assessed.

## Results

We invited 288 patients with a positive HCV antibody to participate in our study, and 270 (96%) agreed to participate. Of these, 265 (98%) completed both surveys. Baseline demographic, clinical, and behavioral characteristics of the sample are reported in **Table 1** [9]. The mean HCV-SS score was 75.6 (SD, 19.1). A total of 212 (80%) participants answered ≥9 items correctly (median number of correct responses, 9 [IQR, 9–10]), equivalent to at least 82% correct answers across all of the knowledge items. The frequency of correct responses to knowledge items did not significantly differ between HIV/HCV-coinfected and HCV-monoinfected participants (median number of correct responses, 10 versus 9; p = 0.30). In the linear regression model evaluating the association between overall HCV knowledge and HCV-SS scores, the interaction between HIV status and knowledge score was non-significant (p = 0.43), so results were not stratified by HIV.

Greater number of correct responses on the NHANES HCV knowledge survey was associated with significantly higher HCV-SS scores in both unadjusted (β, 2.34 [95% CI, 0.51–4.17], p = 0.013) and fully adjusted (β, 2.39 [95% CI, 0.47–4.30], p = 0.015) analyses. For every correctly answered knowledge item, mean HCV-SS score increased by 2.3 points (**Table 2**).

**Table 2:** Association between knowledge of hepatitis C virus and hepatitis C-related stigma.

	Unadjusted		Fully adjusted*	
Model	Beta (95% CI)	P-value	Beta (95% CI)	P-value
	2.34 (0.51, 4.17)	0.013	2.39 (0.47, 4.30)	0.015
Sensitivity analysis excluding participants who responded “True” to all knowledge items (n = 261)	2.93 (1.14, 4.72)	0.001	2.74 (0.88, 4.61)	0.004

*Adjusted for age, race, ethnicity, education level, stage of HCV management, time since diagnosis, history of injection drug use, and HIV-coinfection.

To account for potential affirmative response bias, we conducted a sensitivity analysis excluding participants (n = 4) who responded “True” to all knowledge items. Among the remaining 261 participants, correct responses on the knowledge survey remained associated with significantly higher HCV-SS scores; for every correctly answered knowledge item, mean HCV-SS score increased by 2.93 ([95% CI, 1.14–4.72]; p = 0.001).

For individual knowledge items, the proportion of participants answering each item correctly ranged from 38.1% to 96.9%. Correct answers to two true items about HCV natural history regarding long-term outcomes (questions 1, 2) and two true items regarding HCV transmission to potential loved-ones (questions 7, 8) were significantly associated with increased HCV-related stigma, while correctly answering one false item about transmission via casual contact (question 10) was associated with decreased stigma (**Table 3**). Overall, for each true item, the mean stigma score was greater among participants who correctly answered the items as ‘true,’ compared to those who incorrectly answered the items as ‘false.’ The highest mean stigma scores were observed when false items were incorrectly answered as true (**Table 3**).

**Table 3:** Association between knowledge items about transmission and natural history of hepatitis C virus and hepatitis C-related stigma.

Knowledge Item	Proportion Correct	Mean HCV Stigma Score		Difference	P > |t|
		Answered Incorrectly	Answered Correctly	(β [95% CI])	
1. If someone is infected with hepatitis C virus, they will most likely carry the virus all their lives.	101 (38.11%)	**72.88**	**79.94**	**7.06**	**0.003**
		**(69.98, 75.78)**	**(76.25, 83.63)**	**(2.37, 11.76)**	
2. Infection with the hepatitis C virus can cause the liver to stop working.	245 (92.45%)	**65.70**	**76.38**	**10.68**	**0.016**
		**(57.36, 74.04)**	**(73.99, 78.76)**	**(1.99, 19.35)**	
3. Someone with hepatitis C can look and feel fine.	255 (96.23%)	67.40	75.89	8.49	0.169
		(55.51, 79.29)	(73.54, 78.24)	(-3.63, 20.61)	
4. You can get hepatitis C by getting a blood transfusion from an infected donor.	249 (93.96%)	74.75	75.62	0.87	0.860
		(65.32, 84.18)	(73.23, 78.01)	(-8.86, 10.60)	
5. You can get hepatitis C by shaking hands with someone who has hepatitis C.	252 (95.09%)	84.38	75.12	-9.27	0.088
		(73.98, 94.79)	(72.75, 77.48)	(-19.94, 1.40)	
6. You can get hepatitis C by kissing someone who has hepatitis C.	190 (71.70%)	78.49	74.42	-4.08	0.118
		(74.16, 82.83)	(71.69, 77.14)	(-9.19, 1.04)	
7. You can get hepatitis C by having sex with someone who has hepatitis C.	226 (85.28%)	**69.82**	**76.56**	**6.74**	**0.042**
		**(63.83, 75.81)**	**(74.07, 79.05)**	**(0.25, 13.23)**	
8. You can get hepatitis C by being born to a woman who had hepatitis C when she gave birth.	212 (80.00%)	**70.38**	**76.87**	**6.49 (0.75,**	**0.027**
		**(65.24, 75.51)**	**(74.30, 79.44)**	**12.23)**	
9. You can get hepatitis C by being stuck with a needle or sharp instrument that has hepatitis C infected blood on it.	257 (96.98%)	66.75	75.84	9.09	0.186
		(53.45, 80.05)	(73.49, 78.19)	(-4.41, 22.59)	
10. You can get hepatitis C by working with someone who has hepatitis C.	241 (90.94%)	**83.33**	**74.79**	**-8.54**	**0.037**
		**(75.69, 90.97)**	**(72.39, 77.21)**	**(-16.55, -0.53)**	
11. You can get hepatitis C by injecting illegal drugs, even if only a few times.	232 (87.55%)	69.97	76.37	6.39	0.072
		(63.44, 76.49)	(73.90, 78.83)	(-0.58, 13.37)	

True items are highlighted in grey; false items are white. For each item, the beta coefficient represents the difference in mean stigma score for a correct answer, compared to an incorrect answer; mean stigma scores for correct and incorrect answers are presented. Bolded results indicate a significant difference in stigma score (P < 0.05) between those who answered the item correctly versus incorrectly.

Abbreviations: CI = confidence interval; HIV = human immunodeficiency virus; HCV = hepatitis C Virus.

## Discussion

Overall, 80% of participants correctly answered >80% of questions about knowledge of HCV transmission and natural history. Knowledge scores were similar between HCV-monoinfected and HIV/HCV-coinfected participants. Contrary to our hypothesis, we found that more knowledge about HCV transmission and natural history was associated with greater HCV stigma. When evaluating items individually, mean stigma scores were higher among participants who answered true items correctly and false items incorrectly. Notably, mean stigma scores were highest when false items regarding how HCV is transmitted (items 5, 6, and 10) were incorrectly answered as true.

There are several potential reasons why knowledge about HCV transmission and natural history might increase HCV-related stigma. Patients with increased HCV-related knowledge may have more awareness of disease stereotypes, social stigmas, and the resulting effects of HCV infection on social and interpersonal interactions, contributing to greater perceptions of HCV-related stigma. Fear of stigmatization may lead patients to manage their diagnosis privately, ultimately reducing opportunities for social support and potentially increasing feelings of shame and internalized stigma [10, 11]. For example, knowledge of the potential for vertical HCV transmission (Item 8) or transmission to a sexual partner (Item 7), may increase perceived stigma while fear of chronic disease (Item 1) and HCV-related liver complications (Item 2) may exacerbate feelings of shame and internalized stigma (self-stigma).

Our findings have important implications for chronic HCV providers and public health practitioners. The introduction of highly-effective, all-oral direct-acting antiviral therapies has revolutionized HCV treatment and the elimination of HCV as a public health threat is now feasible [12, 13]. However, high drug costs, heterogeneity in prior authorization approvals across insurances and state-based Medicaid programs, and experiences of stigma in healthcare continue to be barriers to HCV treatment [14–16]. Most patients with HCV experience some degree of HCV-related stigma [5]. For many patients, reduction of stigma is a motivator to undergo HCV treatment [17, 18]; however, experiences of stigma may persist beyond completion of treatment and achievement of cure [5, 17]. Therefore, psychosocial approaches must complement biological interventions if the public health threat of HCV is to be eliminated. While education to dispel disease-based myths and misinformation has been identified as a key intervention point to reduce disease-related stigma [19, 20], our findings suggest that increasing patient-level knowledge alone may be insufficient to counteract HCV-related stigma. In addition to providing basic education about HCV infection, its transmission routes, and health consequences, clinicians should also spend time inquiring about patients’ beliefs about themselves and their peers as a consequence of their HCV infection status. Allotting time to dispel common misconceptions, such as HCV transmission through casual contact (e.g. shaking hands, working with someone who has HCV) may also help to reduce perceived stigma [20–22]. Clinicians could also consider referring patients who experience stigma and misconceptions related to HCV infection to behavioral specialists and/or peer support groups, which might have alternative approaches to try to ameliorate their feelings of stigma.

This study had several limitations. First, the NHANES HCV knowledge questionnaire was developed nearly 20 years ago and may not reflect the most pertinent HCV knowledge gaps among patients today, such as awareness of highly effective oral antiviral therapies. Second, our findings may not be generalizable to patients who are treated by primary care providers, do not have consistent access to healthcare, or receive care in non-urban settings. At the time of enrollment, all participants in this study were receiving care at urban infectious disease clinics specializing in the management of HCV and HIV infections, which may have contributed to their high HCV knowledge scores. However, we sought to maximize generalizability by including patients at all stages of care, including at their first clinical visit. Third, while we assessed whether patients had a history of injection drug use, we did not ask more detailed questions about current drug use or alternative routes of use (e.g., intranasal use), which limited the depth of our assessment of drug use on the association between stigma and knowledge. Finally, given the greater number of true items in the HCV knowledge questionnaire, there is the potential for affirmative response bias with this measure. Reassuringly, associations were similar in sensitivity analyses excluding participants who answered “True” for all knowledge items. Future studies are needed to update and validate this knowledge scale for patients with HCV.

Our study had several strengths. This is the first analysis to evaluate the relationship between HCV-related knowledge and stigma using a validated HCV stigma scale. Since interventions to reduce stigma often employ educational approaches, understanding the relationship between disease-related knowledge and stigma could facilitate the development of educational interventions to be tailored to the needs of people with HCV. Finally, by administering the questionnaires at several academic and community health clinics across Philadelphia, including within a syringe service program, we were able to include patients who were homeless or living in group-housing and who actively use injection drugs, increasing representativeness.

## Conclusions

In conclusion, we found that increased HCV-related knowledge was associated with greater perceptions of HCV stigma. Knowledge of the risk of chronic disease and liver complications, as well as potential sexual or vertical transmission, may be particularly stigmatizing for patients. Clinicians should consider spending time addressing common misbeliefs about HCV transmission and natural history when educating patients about HCV infection. Correcting such misunderstandings might counterbalance the stigmatizing impact of other disease knowledge, such as HCV-related morbidity and risk of transmission through sex or vertical transmission. Future research should seek to develop and validate a more contemporary instrument assessing knowledge about HCV transmission and natural history to further elucidate the association between HCV-related knowledge and stigma and guide interventions to address patients’ experiences of HCV-related stigma.
